# Improving the Performances of Perovskite Solar Cells via Modification of Electron Transport Layer

**DOI:** 10.3390/polym11010147

**Published:** 2019-01-16

**Authors:** Mao Jiang, Qiaoli Niu, Xiao Tang, Heyi Zhang, Haowen Xu, Wentao Huang, Jizhong Yao, Buyi Yan, Ruidong Xia

**Affiliations:** 1 Key Laboratory for Organic Electronics and Information Displays & Institute of Advanced Materials, National Jiangsu Synergistic Innovation Center for Advanced Materials, Nanjing University of Posts and Telecommunications, 9 Wenyuan Road, Nanjing 210046, China; jm245398846@163.com (M.J.); iamqlniu@njupt.edu.cn (Q.N.); tangxiao20190116@163.com (X.T.); iamhyzhang@163.com (H.Z.); sdwfxhw1021@163.com (H.X.); wthuang7@163.com (W.H.); 2 Microqanta Semiconductor Company, 998, West Wenyi Road, Hangzhou 311121, China; jizhong.yao@microquanta.com (J.Y.); buyi.yan@microquanta.com (B.Y.)

**Keywords:** perovskite solar cells, surface morphology, PCBM, doping

## Abstract

The commonly used electron transport material (6,6)-phenyl-C61 butyric acid methyl ester (PCBM) for perovskite solar cells (PSC) with inverted planar structures suffers from properties such as poor film-forming. In this manuscript, we demonstrate a simple method to improve the film-forming properties of PCBM by doping PCBM with poly(9,9-dioctylfluorene-co-benzothiadiazole) (F8BT) as the electron transport layer (ETL), which effectively enhances the performance of CH_3_NH_3_PbI_3_ based solar cells. With 5 wt % F8BT in PCBM, the short circuit current (J_SC_) and fill factor (FF) of PSC both significantly increased from 17.21 ± 0.15 mA·cm^−2^ and 71.1 ± 0.07% to 19.28 ± 0.22 mA·cm^−2^ and 74.7 ± 0.21%, respectively, which led to a power conversion efficiency (PCE) improvement from 12.6 ± 0.24% to 15 ± 0.26%. The morphology investigation suggested that doping with F8BT facilitated the formation of a smooth and uniform ETL, which was favorable for the separation of electron-hole pairs, and therefore, an improved performance of PSC.

## 1. Introduction

Organometal halide perovskite-based solar cells (PSCs) have attracted increasing attention throughout the world because of their high power conversion efficiency (PCE), low cost and easy fabrication techniques [1,2,3]. The excellent device performance can be owed to the unique properties of perovskite light harvesters such as strong absorption coefficients, appropriate direct band gaps, high charge carrier mobility and exceptional long charge carrier diffusion lengths [4,5]. After the first report in 2009 [6], the PCE of PSC has seen rapid improvement, exceeding 20% in recent reports [7,8] and demonstrating its great potential for the future low-cost photovoltaic technology.

Planar structure PSC has great potential because of its simple solution-based fabrication technique. According to the order of preparation, the planar structure can be divided into two types: n-i-p and p-i-n [9,10]. For a PSC with a p-i-n structure, organic materials poly(3,4-ethylenedioxythiophene) polystyrene sulfonate (PEDOT:PSS) and (6,6)-phenyl-C61 butyrie acid methyl ester (PCBM) were usually used as the hole and electron transport layers (HTL and ETL) [11,12]. Both of them can be fabricated with solution methods at room temperature. Thus, the p-i-n structure is compatible with the flexible devices and the upscaling of PSC technology, which are the future development directions of optoelectronic devices [13,14]. Except for the conventional PSC based on organometal halide perovskites, the p-i-n structure was also the common PSC configuration based on Pb-free perovskites, which has recently attracted tremendous interest around the world [15].

In a p-i-n PSC device, PCBM was usually deposited on the surface of the perovskite layer as the ETL. It can enhance electron and hole separation by smoothing the perovskite surface and passivating surface traps [16,17]. Thus, PCBM plays a very important role in p-i-n PSC. However, PCBM is far from perfect as the ETL because of the poor film-forming, general electron mobility and energy level mismatch between the lowest unoccupied molecular orbital (LUMO) (−3.9 eV) and the work function of the commonly used Ag electrode (4.3 eV) [18]. Hence, optimization of the PCBM layer is an effective approach to improving the performance of PSC [19,20,21,22,23,24,25,26,27,28,29]. For example, by incorporating a hole blocking layer, such as TiO_2_ [20], LiF [21], PN_4_N [22], zwitterion/LiF [23], perylene-diimide [24] and PFN [25], the efficiency of p-i-n PSC was greatly enhanced. An alternative method to optimizing the PCBM layer is doping. The doping strategy is not only effective but also simple in regards to fabricating techniques. On the one hand, various materials were selected in order to improve the electron mobility of PCBM, such as reduced graphene oxide [26], fluorene-based polyelectrolytes [27], graphdiyne [28] and oleamide [29]. On the other hand, Zhao et al. reported a PCE increase from 9.56% to 10.68% by doping PCBM with polystyrene (PS) due to the improved film-forming properties of the ETL [30]. However, since PS is an insulator, the amount of PS in PCBM should be controlled very carefully to avoid significant reduction of the short circuit current (J_SC_). Therefore, this doping method can be optimized by using dopants with good film-forming properties and electrical conductivities.

Fluorene-based polymers such as poly(9,9-dioctyfluorene) (PFO) and poly(9,9-dioctylfluorene-*co*-benzothiadiazole) (F8BT) are commonly used polymers in organic optoelectronic devices [31,32]. They are semiconductors with excellent film-forming properties. Therefore, it is natural to investigate the influence of PFO and F8BT, as dopants in PCBM, on the performances of PSCs, which was carried out in this work. Experimental results showed that the surface roughness of ETL was reduced. The improved film uniformity was favorable for the separation of electron-hole pairs. With 5 wt % F8BT in PCBM as ETL, the short circuit current (J_SC_), fill factor (FF) and PCE of PSC all increased, from 17.21 ± 0.15 mA·cm^−2^, 71.1 ± 0.07% and 12.6 ± 0.24% to 19.28 ± 0.22 mA·cm^−2^, 74.7 ± 0.21% and 15 ± 0.26%, respectively. The influence of PFO in the performances of PSCs was also studied. The charge carrier dynamics were analyzed as well.

## 2. Materials and Methods

### 2.1. Materials

PFO and F8BT were synthesized in South China University of Technology according to Reference [33]. CH_3_NH_3_PbI_3_ (MAI), PbI_2_, PCBM and 2,9-dimethyl-4,7-diphenyl-1,10-phenanthroline (BCP) were purchased from Xi’an Polymer Light Technology Corp. (Xi’an, China). Dimethyl sulfoxide (DMSO), chlorobenzene and Dimethyl Formamide (DMF) were all purchased from Shanghai Aladdin Bio-Chem Technology Co., Ltd. (Shanghai, China) and used as received. NiO_x_ nanoparticles were synthesized in our lab according to Reference [34].

MAPbI_3_ precursor solution was prepared by dissolving 1.4 mmol MAI and 1.4 mmol PbI_2_ in 1 mL GBL and DMSO (7:3/*v*:*v*). Before spin-coating, the MAPbI_3_ precursor solution was stirred at 65 °C for 12 h in a N_2_ atmosphere glove box.

### 2.2. Device Fabrication

The indium tin oxide (ITO)-coated glass substrates were cleaned sequentially in detergent, deionized water, acetone and ethanol under sonication for 20 min, respectively. After being dried by N_2_ flow, 4 min of ozone plasma at a power of 70 W was applied to remove any organic residues. Immediately after, NiO_x_ nanoparticle solution was spin-coated onto the ITO glass at 4000 rpm for 30 s and then baked at 130 °C for 20 min. The substrate was then transferred into a N_2_ protected glovebox with the contents of water and oxygen less than 1 ppm. Following this, the MAPbI_3_ precursor solution was spin-coated. Specifically, the spin-coating process was composed of two stages: 900 rpm for 15 s and then 4000 rpm for 25 s. To perform toluene washing during the spin-coating of MAPbI_3_ precursor solution at a delay time of 15 s from the beginning of the second stage, 400 μL of toluene was dripped. After thermally annealing at 100 °C for 10 min, the mixed PCBM solution with F8BT or PFO in chlorobenzene (20 mg/mL) was spin-coated at a speed of 1000 rpm for 20 s. A control device was also fabricated with PCBM as the ETL. Then, the substrates were annealed at 70 °C for 40 min. Finally, BCP and Ag were thermally evaporated in sequence at a basic pressure of 4 × 10^−4^ Pa. The active area was 0.077 cm^2^ defined by a shadow mask.

To check the reproducibility of PSCs, multiple batches of the devices were made. The average values in performances were obtained accordingly, which were then used to evaluate the experimental error.

### 2.3. Device Characterization

The current density–voltage (J–V) characterization of PSC was carried out using a Keithley 2400 source meter under a simulated AM 1.5 illumination (100 mW·cm^−2^, NewPort, 94043A SOLAR SIM, Irvine, CA, USA) at a scan rate of 200 mV·s^−1^. The incident-photon-to-current efficiency (IPCE) measurement was performed through an Enlitech QE-R Quantum Efficiency Measurement System (QE-R3018, Taiwan, China). The crystal structures of the MAPbI_3_ films were characterized by using a Bruker D8 ADVANCE X-ray diffraction (XRD) device (Karlsruhe, Germany). The morphology of the MAPbI_3_ layer was obtained using a Hitachi S-4800 field emission scanning electron microscope (SEM, Tokyo, Japan) and a Bruker atomic force microscope (AFM). The thicknesses of the NiO_x_, MAPbI_3_ and PC_61_BM films were determined using a Bruker DektakXT Stylus Profiler. The photoluminescence (PL) spectra were measured using an Edinburgh FLS980 fluorescence spectrophotometer (Livingston, UK) with an excitation at 550 nm. The impedance characteristics of the devices were measured via a Wayne Kerr 6500B analyzer (London, UK). 

## 3. Results

### 3.1. Device Performance 

PFO and F8BT are commonly used polymers in organic optoelectronics devices, which have good film-forming properties. Especially, F8BT is one of the few polymers with better electron mobility than hole mobility [31], whereas PFO is a typically hole-dominated polymer [32]. Thus, PCBM was doped with PFO and F8BT as the ETL, respectively, to investigate the influence of film-forming and charge carrier transport properties of the ETL on the performances of PSCs.

**Figure 1:**
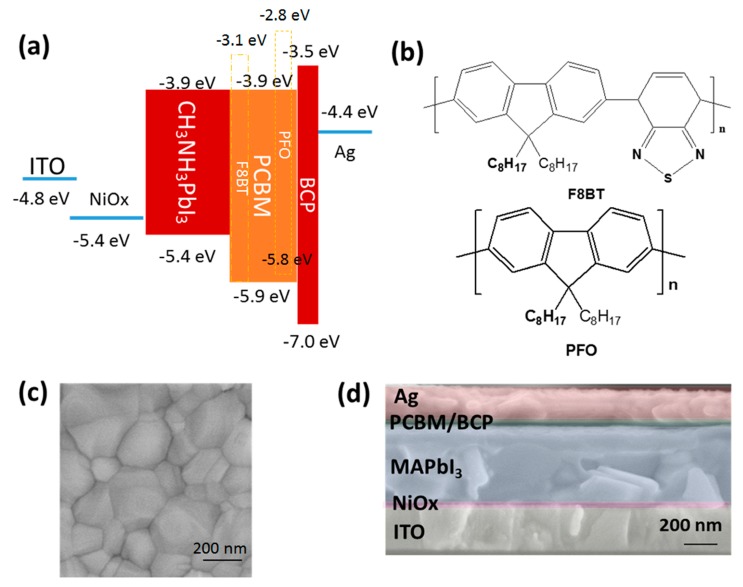
(**a**) The schematic diagram of the device architecture and energy levels of the device layers, (**b**) the chemical structure of poly(9,9-dioctyfluorene) (PFO) and poly(9,9-dioctylfluorene-co-benzothiadiazole) (F8BT), (**c**) the top SEM image of perovskite film and (**d**) the sectional SEM image of perovskite solar cells (PSC).

The device configuration of PSC incorporating the energy level of each layer is pictured in Figure 1a, wherein the energy level values were taken from the literatures [35,36,37]. Figure 1b shows the chemical structure of PFO and F8BT used in this study. MAPbI_3_ was deposited by using a one-step solution method via spin-coating the perovskite precursor solution of MAI: PbI_2_ (1:1) in GBL and DMSO (7:3/*v*:*v*). Mirror-like dark-brown MAPbI_3_ film was obtained after thermal annealing at 100 °C for 10 min. The perovskite crystal size was evaluated from the scanning electron microscope (SEM) image as shown in Figure 1c, which was about 200 nm in diameter. The cross-sectional SEM image (Figure 1d) shows the pinhole-free perovskite film fully covered on the NiO_x_ substrate.

**Figure 2:**
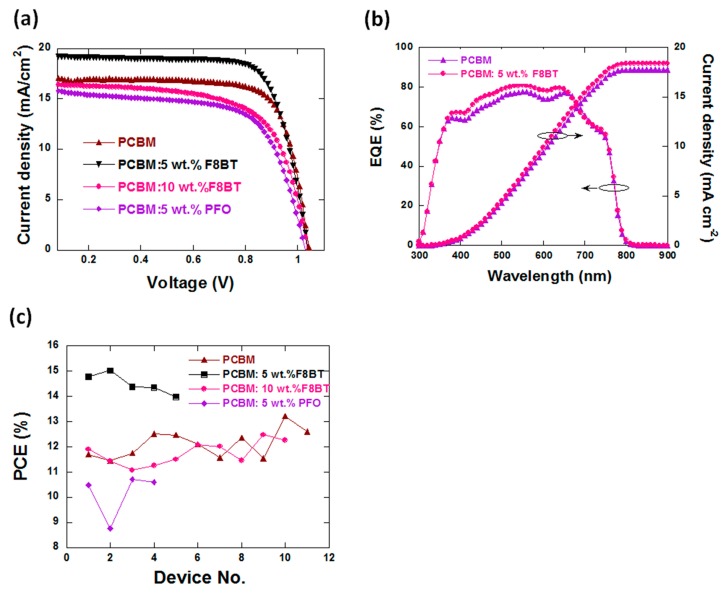
(**a**) Photocurrent density (J)–voltage (V) curves of the devices (under 100 mW·cm^−2^ AM 1.5 illumination) with different electron transport layers (ETL), (**b**) EQE data and integrated J_SC_ curves (**c**) and the PCE statistics of PSCs.

Figure 2a shows the current density–voltage (J–V) curves of PSCs as a function of the weight ratios of PCBM to F8BT or PFO. The detailed performance parameters of the devices are summarized in Table 1. The experimental error values were obtained by minus the average value and then divided by 2. The control device based on pure PCBM ETL had a PCE of 12.6 ± 0.24% with a V_OC_ of 1.03 ± 0.01 V, a J_SC_ of 17.21 ± 0.15 mA·cm^−2^ and a FF of 71.1 ± 0.07%. After doping PCBM with 5 wt % F8BT, the device demonstrated a clear increase of PCE to 15 ± 0.26%, with a V_OC_ of 1.04 ± 0.01 V, a J_SC_ of 19.28 ± 0.22 mA·cm^−2^ (12% enhancement) and a FF of 74.7 ± 0.21%. V_OC_ was almost unchanged after doping. However, the enhancement of J_SC_ was significant after doping with F8BT in PCBM, which caused the increase of PCE. The increase of J_SC_ was further justified by the IPCE data as shown in Figure 2b. The integrated J_SC_ values from the IPCE spectrum were 17.77 and 18.44 mA·cm^−2^ for the control device and the PCBM: 5 wt % F8BT-based device, respectively, which were close to the J_SC_ values from the J–V scan. When increasing the F8BT doping ratio to 10 wt.%, the PCE of the device began to decrease with loss in J_SC_. It should be noted that the typical structure of p-i-n type PSC includes ITO/HTL/perovskite/ETL/cathode. The performance of PSC can be influenced by each layer and the interfaces between them. Therefore, there are many factors that can be considered to improve the performance. In this work, to emphasize the effect of the film-forming property of PCBM on the performance of PSC, a basic device structure was used. That is why the performance in this manuscript was moderate compared with the reports. We except better performance with higher PCE if combining with other optimizing approaches.

In the case of doping PCBM with 5 wt % PFO, the PCE of PSC decreased to 10.8 ± 0.27% because of the decrease of J_SC_ and FF to 16.3 ± 0.24 mA·cm^−2^ and 64 ± 0.98%, respectively. A possible explanation is that PFO is a hole-dominated material with a very low electron mobility [38]. The doping of PCBM with PFO is unfavorable to electron transporting and the collection of ETL. Therefore, PFO was not suitable to be used as a dopant in PCBM as the ETL of PSCs. The PCE value statistics of PSCs using multiple batches are shown in Figure 2c.

We noted that the optimal F8BT doping ratio was 5 wt % in our study, whilst doping with only 1.5 wt % PS caused a decrease of J_SC_ as reported recently [30]. Therefore, the content of PS has to be strictly controlled at a very low concentration, which limits the improvement of the PCE of the device (from 9.56% to 10.68%, a 11.7% enhancement). However, F8BT is a semiconductor with good electron transport properties [39], which made it possible to dope PCBM with a high content of F8BT, leading to a more effective improvement of the PCE (19% enhancement for 5 wt % doping) as shown in Figure 2a and Table 1.

### 3.2. Film Properties

**Figure 3:**
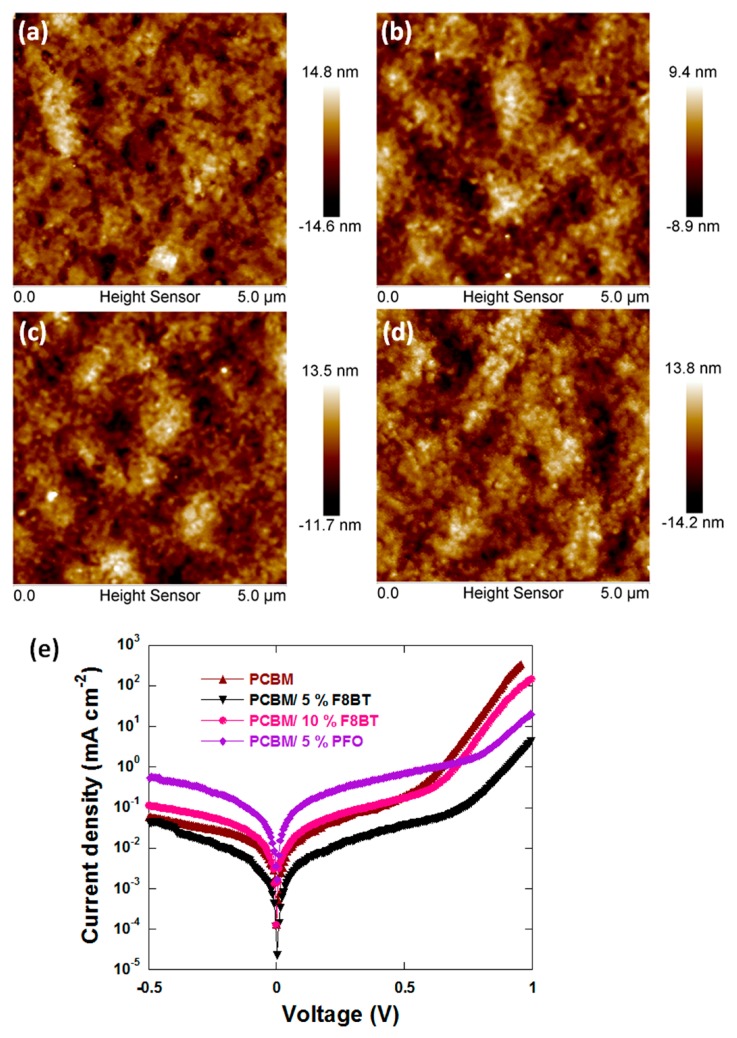
Atomic force microscopy (AFM) images of (**a**) PCBM, (**b**) PCBM: 5 wt % F8BT, (**c**) PCBM: 10 wt % F8BT and (**d**) PCBM: 5 wt % PFO, and (**e**) J–V characteristics of the device under dark with 0 wt % and 5 wt % F8BT.

To gain more insights into the PCBM: F8BT films, atomic force microscopy (AFM) was used to examine the impact of the F8BT additive on the film property of the PCBM layer. The 5 μm × 5 μm AFM topography images of PCBM films (with or without F8BT) are shown in Figure 3. The pure PCBM layer had a root mean square roughness (rms) value of 3.9 nm, which decreased to 2.7 nm and 3.58 nm with the addition of 5 wt % and 10 wt % F8BT, respectively, resulting in a smoother surface. This can be attributed to the good solubility of F8BT in chlorobenzene and its good film-forming properties [40,41]. The ETL layer with good uniformity and smoother surface on the perovskite photoactive layer will more effectively prevent undesirable electrons-holes recombination between the metal electrode and the perovskite layer. This led to the reduction of the dark current, as shown in Figure 3e. Eventually the device performances increased.

**Figure 4:**
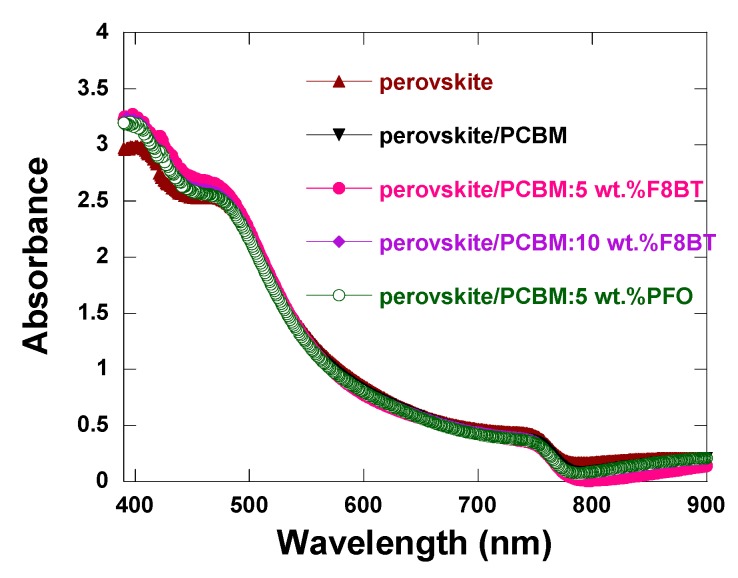
Ultraviolet–visible absorption spectra of perovskite, perovskite/PCBM, perovskite/PCBM: x wt % F8BT (x = 5, 10) and perovskite/PCBM: 5 wt % PFO.

For a photovoltaic device, efficient light absorption of perovskite was the primary factor for high solar cell performance. Thus, the light absorption of the films of perovskite and perovskite /ETL were examined, as shown in Figure 4. It can be seen that in the band of 460–700 nm, absorbance intensities of all samples were almost the same. In the band of 390–460 nm, the absorbance intensity slightly increased after depositing PCBM or PCBM doped with F8BT (or PFO) layer due to the absorption of PCBM, F8BT and PFO.

### 3.3. Charge Carrier Dynamic

**Figure 5:**
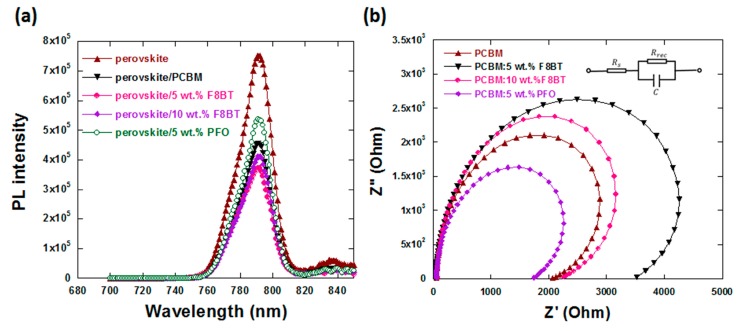
(**a**) The PL spectra of perovskite and perovskite/ETL double layered films and (**b**) Nyquist plots of PSCs with different ETLs.

The PL spectra in Figure 5a peaked at 780 nm were originated from MAPbI_3_. When in contact with PCBM, the light intensity of perovskite/PCBM decreased because of the transfer of electrons from perovskite to PCBM. It decreased further when PCBM was doped with F8BT, indicating the more effective electron transfer [42]. However, when in contact with PCBM doped with PFO, the PL intensity of MAPbI_3_ increased, indicating a worse electron transfer from perovskite to ETL.

To further understand the effect of F8BT or PFO on the device performances, electrochemical impedance spectroscopy (EIS) measurements for the PSCs based on different ETLs was carried out with their Nyquist plots shown in Figure 5b. The arc in the low-frequency region of the Nyquist plots is associated with the recombination process within the PSCs. It can be described by using the recombination resistance (R_rec_). Higher R_rec_ values represent less recombination and more effective dissociation of carriers at the interfaces [43]. After doping PCBM with 5 wt % F8BT, the R_rec_ values of PSCs increased from 157 Ω·cm^2^ to 272 Ω·cm^2^, indicating more effective dissociation of carriers at the interfaces of PSCs, which is good for the performances of PSCs. 

### 3.4. Energy Level Matching

From the above experimental results, we can see that the doping of PCBM with F8BT resulted a smoother surface, which was favorable for the separation of electron-hole pairs. However, the PCE of PSC based on PCBM: 10 wt % F8BT decreased compared with the control device. That might be due to the fact that the lowest unoccupied molecular orbital (LUMO) of F8BT was higher than that of PCBM, which is bad for the collection of electrons [44]. In addition, though F8BT is an electron-dominated material, its electron mobility was lower than that of PCBM [39,45]. Hence, when the content of F8BT in PCBM was higher than 5 wt %, the J_SC_ of PSC decreased even though the surface roughness of ETL had improved.

## 4. Conclusions 

Highly efficient PSCs were fabricated by doping PCBM with F8BT as the ETL, which led to significant improvements of J_SC_ and FF, thus a great enhancement of PCE. With 5 wt % F8BT in PCBM as the ETL, a 19% enhancement of the PCE of PSC was achieved. The morphology study shows that doping with F8BT improved the uniformity of the ETL, which is favorable for the separation of electron-hole pairs. Therefore, the collection of electrons was improved. Our method has shown to be an effective and simple way to realize highly efficient PSCs.

## Figures and Tables

**Table 1:** Summary of detailed performance parameters of PSCs.

ETLs Used	V_OC_ (V)	J_SC_ (mA·cm^−2^)	FF ^a^ (%)	PCE ^b^ (%)
PCBM ^c^	1.03 ± 0.01	17.21 ± 0.15	71.1 ± 0.07	12.6 ± 0.24
PCBM: 5 wt % F8BT	1.04 ± 0.01	19.28 ± 0.22	74.7 ± 0.21	15 ± 0.26
PCBM: 10 wt % F8BT	1.02 ± 0.01	17.13 ± 0.28	71.5 ± 0.63	12.48 ± 0.37
PCBM: 5 wt % PFO	1.03 ± 0.02	16.3 ± 0.24	64 ± 0.98	10.8 ± 0.27

^a^ Fill factor; ^b^ Power conversion efficiency; ^c^ (6,6)-phenyl-C61 butyric acid methyl ester.
